# Stereotactic ablative radiotherapy (SABR) as primary, adjuvant, consolidation and re-treatment option in pancreatic cancer: scope for dose escalation and lessons for toxicity

**DOI:** 10.1186/s13014-018-1138-3

**Published:** 2018-10-19

**Authors:** Christy Goldsmith, P. Nicholas Plowman, Melanie M. Green, Roger G. Dale, Patricia M. Price

**Affiliations:** 1 Guys and St Thomas’ NHS Foundation Trust, London, UK; 2 The London CyberKnife Centre, The Harley Street Clinic, 81 Harley Street, London, W1G 8PP UK; 3 St. Bartholomew’s Hospital, London, UK; 4 Department of Surgery and Cancer, Imperial College London, London, UK

**Keywords:** Stereotactic ablative radiotherapy (SABR), Cyberknife®, Pancreatic cancer, Radiobiology, Dose escalation, Local control, Toxicity, Survival

## Abstract

**Background:**

Stereotactic ablative radiotherapy (SABR) offers an alternative treatment for pancreatic cancer, with the potential for improved tumour control and reduced toxicity compared with conventional therapies. However, optimal dose planning and delivery strategies are unelucidated and gastro-intestinal (GI) toxicity remains a key concern.

**Methods:**

Patients with inoperable non-metastatic pancreatic cancer who received CyberKnife® SABR (18–36 Gy) in three fractions as primary, adjuvant, consolidation or re-treatment options were studied. Patient individualised planning and delivery variables were collected and their impact on patient outcome examined. Linear-quadratic (LQ) radiobiology modelling methods were applied to assess SABR parameters against a conventional fractionated radiotherapy schedule.

**Results:**

In total 42 patients were included, 37 (88%) of whom had stage T4 disease. SABR was used > 6 months post-primary therapy to re-treat residual disease in 11 (26.2%) patients and relapsed disease in nine (21.4%) patients. SABR was an adjuvant to other primary therapy for 14 (33.3%) patients and was the sole primary therapy for eight (19.0%) patients. The mean (95% CI) planning target volume (PTV), prescription isodose, percentage cover, minimum dose to PTV and biological effective dose (BED) were 76.3(63.8–88.7) cc, 67.3(65.2–69.5)%, 96.6(95.5–97.7)%, 22.3(21.0–23.6) Gy and 50.3(47.7–53.0) Gy, respectively. Only 3/37 (8.1%) patients experienced Grade 3 acute toxicities. Two (4.8%) patients converted to resectable status and median freedom-from-local-progression (FFLP) and overall survival (OS) were 9.8 and 8.4 months, respectively. No late toxicity was experienced in 27/32 (84.4%) patients; however, four (12.5%) patients — of whom two had particularly large PTV, two had sub-optimal number of fiducials and three breached organ-at-risk (OAR) constraints—showed Grade 4 duodenal toxicities. Longer delivery time, extended treatment course and reduced percentage coverage additionally associated with late toxicity, likely reflecting parameters typically applied to riskier patients. Larger PTV size and longer treatment course associated with OS. Comparator regimen LQ modelling analysis indicated 50% of patients received minimum PTV doses less potent than a conventional radiotherapy regimen, indicating scope for dose escalation.

**Conclusion:**

The results demonstrate the value of SABR for a range of indications in pancreatic cancer. Dose escalation to increase BED may improve FFLP and OS in inoperable, non-metastatic disease: however concomitant enhanced stringency for duodenal protection is critical, particularly for patients where SABR is more challenging.

## Background

Pancreatic cancer is characterised by debilitating symptoms caused by local disease advance, high rates of metastatic progression and dismally close incidence and mortality rates. Median overall survival (OS) from diagnosis for all patients is less than 5 months, ~ 20% of patients overall survive more than 1 year and ~ 5% survive more than 5 years [1–4]. Standard treatment options include single or multi-agent chemotherapy or chemoradiotherapy. For the small minority of patients diagnosed with earlier stage resectable disease, radical surgical resection offers the best chance of long-term survival with reported 5-year survival rates of ~ 20% [5]. However, the disease is typically diagnosed at more advanced stages: ~ 45% of patients present with metastatic disease that has a median OS of ~ 2–5 months, and ~ 30% present with inoperable localized or locally advanced pancreatic cancer (LAPC) that has an intermediate status and prognosis [1–4]. For the latter subset of patients, conversion to resectability currently offers the best outcome [5–7]: nevertheless, median OS reported in LAPC randomised trials remains poor at 7–15 months [8–10].

Modern conformal radiotherapy has a central role in pancreatic cancer disease control to: 1) palliate symptoms and prevent local progression that causes morbidity and may be causal of death [11]; 2) increase the chances of achieving secondary resectability [6, 7]; and 3) extend median survival time [12–15]. However, conventional chemoradiotherapy regimens can be long (~ 5.5 weeks) and arduous with only a modest or no overall survival gain [16]. Furthermore, the radiation doses that can be delivered safely are limited by toxicity to adjacent abdominal structures such that local control rates can be low [8, 15, 17, 18], repeat treatment upon relapse is inhibited, and treatment toxicity may be high and exacerbated by concomitant chemotherapeutics [14, 19–22].

Stereotactic ablative body radiotherapy (SABR) may be used as an alternative, adjunct, consolidation or re-treatment option to conventional therapies in pancreatic cancer. High dose radiotherapy can be delivered in conveniently few fractions, with rapid dose fall-off outside the delineated tumour volume [23, 24]. This offers the potential for increased local tumour control and reduced toxicity, and additionally introduces the valuable possibility of tumour re-irradiation. Due to the highly conformal target volumes delineated, the high doses delivered and the proximity of adjacent radiosensitive organs-at-risk (OAR — duodenum, stomach, small bowel, liver, kidneys and spinal cord), accurate on-treatment tumour targeting is imperative.

Previous studies have pioneered the use of SABR in pancreatic cancer in various regimens, including as a single boost adjunct to chemotherapy or chemoradiotherapy or for re-treatment following local failure [20–22, 25–37]. The majority have shown relative efficacy for local control with reasonable toxicity, as well as benefits such as convenience and good pain relief [37]. Furthermore, receipt of SBRT compared with conventionally fractionated radiotherapy has shown significantly improved OS for locally advanced disease [38]. However, comparisons, interpretations and treatment optimisation of SABR in pancreatic cancer have been difficult as different planning, delivery and dosimetry methodologies have been used. As a result, local control has been reported as excellent in some studies [22, 25, 29, 31], but not in others [20, 21] and toxicity has been reported to be low in some studies [26, 33], but significant or unacceptable in others [20–22, 25, 28, 36, 39]. With the aim of increasing understanding and enabling treatment improvements for inoperable, non-metastatic pancreatic cancer, in our real-world series of patients we sought to: 1) review patient outcomes following SABR treatment for a range of indications 2) comprehensively examine the contribution of patient, planning, delivery and radiobiological variables upon the toxicity and efficacy of fractionated SABR and 3) use radiobiological modelling to compare SABR treatment to a conventional comparator regimen.

## Methods

### Patients and data collection

Pancreatic cancer patients referred to The CyberKnife® Centre at The Harley Street Clinic for SABR treatment from March 2009 – October 2012 were included. For comparative radiobiological and outcome purposes, only patients with inoperable status and no detectable metastases (i.e. those with localized or locally advanced disease that was unresectable, who had a prognosis intermediate to resectable and metastatic disease), who were treated in three fractions, were studied. Patient, tumour, treatment planning and delivery parameters were collected at the time of treatment. Patients were longitudinally followed and toxicity and outcome data were acquired at follow-up clinic appointments.

### Treatment

SABR was delivered with the CyberKnife™® robotic radiosurgery system (Accuray® Corporation, Sunnyvale, CA, USA) [40]. In the majority of cases, following percutaneous implantation of gold fiducial tumour markers (Cybermark, CIVCO Medical Solutions, Iowa, USA) under computed tomography (CT) or endoscopic ultrasound (EUS) guidance, the Synchrony® (Accuray® Corporation, Sunnyvale, CA, USA) near real-time kilovoltage imaging system was used to achieve dynamic radiation delivery adaptive to patient and tumour movement.

Planning CT scans were performed at least one week after fiducial placement. Patients were advised to drink 300 mls of cold water 30 mins before scanning to promote duodenal filling. Patients were scanned supine in the treatment position and immobilised with an individualised Vac-Lok cushion (Civco Medical Solutions, Iowa, USA) to support the arms and upper torso. Knee and ankle supports were used where needed. A non-contrast CT was performed in all patients and unless contra-indicated by poor renal function, a contrast CT scan was also performed to aid target definition following intravenous administration of 100 mls Omnipaque. Scans were performed in mid-breath hold and were acquired with 1.25 mm slice thickness. The field-of-view encompassed the entire circumference of the body contour with coverage from 15 cm above the most superior fiducial to 15 cm below the most inferior fiducial. Target localisation was performed on the CyberKnife® MultiPlan® Treatment Planning System (Accuray Inc. Sunnyvale, CA, USA). Treatment was planned on the non-contrast CT scan which was fused with the contrast CT to aid target localisation. Where available, pre-treatment combined 18-fluorodeoxyglucose positron emission tomography and CT (^18^F-FDG-PET/CT) scan was also fused with the planning CT. Gross tumour volume (GTV) was outlined by the treating clinician, reviewed by a radiologist where appropriate and expanded with an isocentric margin of 2–3 mm to form the planning target volume (PTV).

In two palliative cases where fiducial tracking was not performed, patients were planned and monitored with an internal tumour volume (ITV) and XSight® Spine Tracking (Accuray Corporation, Sunnyvale, CA, USA) approach. Planning CT scans were acquired in free-breathing, maximal inspiration and maximal expiration respiratory phases. An internal target volume (ITV) was constructed to incorporate maximal GTV excursion with respiration and XSight® Spine tracking was used throughout treatment to assure reproducible patient body anatomy positioning.

For all patients, the duodenum, stomach, small bowel, liver, kidneys and spinal cord were outlined and treated as OAR. The duodenum was considered the primary OAR and constraints were applied as previously published [41, 42]. The preferred OAR dose constraints for three fraction SABR (applied when feasible) are given in Table 1. Tighter constraints were applied if previous radiotherapy had been given. Patients were prescribed 18–36 Gy in three fractions, with total dose influenced by the proximity of dose-limiting OAR (primarily duodenum and stomach) and any prior radiotherapy received. The prescription isodose line was chosen to provide optimum coverage of the target volume whilst respecting OAR constraints. Tumour definition, normal tissue constraints and final treatment plan were approved by the consultant radiation oncologist and attending medical physicist.

**Table 1:** OAR dose constraints applied for three fraction SABR in this study

	D0.035 cc	D0.1 cc	D10.0 cc	D5.0 cc	V12 Gy	V15 Gy	V21 Gy
Duodenum	≤ 22.2 Gy	–	≤ 11.4 Gy	≤ 16.5 Gy	–	–	–
Stomach	≤ 22.2 Gy	–	≤ 16.5 Gy	–	–	–	–
Small Bowel	≤ 25.2 Gy	–	–	≤ 17.7 Gy	–	–	–
Liver	–	–	–	–	–	≤ 50%	≤ 30%
Kidneys (separate)	–	–	–	–	≤ 25%	–	–
Kidneys (together)	–	–	–	–	–	≤ 35%	–
Spinal cord	≤ 21.0 Gy	≤ 18.0 Gy	–	–	–	–	–

Note: For all pancreatic patients treated with ≤ 30 Gy in three fractions, we have since revised our duodenal D0.035 cc constraint to the higher limit of 24 Gy, but additionally now use D1.0 cc < 31.4 Gy

Prior to each treatment fraction, patients were pre-medicated with prophylactic ondansetron 4 mg and metoclopramide 10 mg orally (unless contra-indicated). Patients drank 300 mls of water and set-up was performed as per CT planning. A non-contrast mid-breath hold CT scan was performed on Day 1 for patients with implanted fiducial markers to assess fiducial stability. This scan was fused with the planning CT and was assessed on the MultiPlan® System (Accuray Corporation, Sunnyvale, CA, USA), prior to radiation delivery to ensure that fiducial placement was maintained. Once the CyberKnife® physicist and treating physician were satisfied that the fiducial arrangement on the Day 1 CT matched that of the planning CT, the GTV and PTV contours were overlaid on the Day 1 CT to check contours appropriately covered the tumour target, with no discernible differences to adjacent OAR (e.g. stomach/duodenal filling or tumour growth) impacting target coverage. Following this check, most patients were treated on 3 consecutive weekdays; in all cases treatment was completed within 7 days.

### Follow-up

Patients were clinically reviewed 3-months after treatment, then at 6-month intervals thereafter when post-treatment CT scans were acquired. Toxicity was assessed using Common Terminology Criteria for Adverse Events version 3 (CTCAEv3). Local and distant failures were determined by an independent reporting radiologist and the treating physician using radiological information. Local control was defined as stable or decreased tumour size, local failure was defined by an increase in tumour size and distant failure was defined as the appearance of new lesions on CT or PET scan.

### BED calculation and radiobiological modelling

The biological effective dose (BED) received by each patient was calculated simply as:

$$ \mathrm{BED}=\mathrm{D}\times \left[1+\frac{d}{\frac{\alpha }{\beta }}\right] $$

where *D* is the total dose delivered, d is the dose per fraction and α/β is the assumed fractionation factor. For pancreatic tumour, the radiobiological assumption was α/β = 10 Gy. For normal tissues the radiobiological assumption was α/β = 3 Gy. Comparison between SABR and conventional radiotherapy schedules was carried out using the linear-quadratic (LQ) model modified to account for tumor repopulation. The conventional radiotherapy comparator schedule used was 50.4 Gy in 28 fractions over a total period of 5.5 weeks (37 days). For the calculation of iso-effective SABR schedules a repopulation correction was applied to the BED calculation formula [43, 44], where for pancreatic tumour the radiobiological parameter assumptions used were: doubling time = 42 days, k = 0.5 Gyday^− 1^ and repopulation was assumed to be operative throughout the entire treatment. As the majority of SABR treatments were completed within 3 days, a fixed repopulation correction of 3 × 0.5 Gy = 1.5 Gy was used.

### Statistical analysis

The Chi-squared test or ANOVA analysis was used to determine the association between planning and delivery variables and toxicity. Kaplan-Meier analysis was used to calculate freedom-from-local-progression (FFLP), progression-free-survival (PFS) and overall survival (OS), beginning from the start of SBRT treatment and censoring patients lost to follow-up. Log-rank analysis was used to assess the impact of factors on FFLP and OS. The Cox proportional hazard model, adjusted for all variables, was used to assess the impact of multi-variate factors upon FFLP and OS.

## Results

### Patient, tumour and treatment purpose characteristics (Table 2)

A total of 42 patients were included, with median (range) patient age of 64 (42–85) years. The majority of patients had stage T4 disease located in the head of the pancreas. The reported reasons for SABR as the sole primary treatment (8 patients) were poor performance status (PS), co-morbidity and patient choice. SABR was considered as an adjuvant when it was used within 6 months of standard therapies (14 patients). SABR was used 6 months–3 years after primary therapy as a consolidation or re-treat option for residual or relapsed local disease in 20 patients.

**Table 2:** Patient, tumour and treatment characteristics (*N* = 42 patients)

Gender	Male	N = 16 (38.0%)
	Female	N = 26 (62.0%)
Tumour stage	T4	N = 37 (88.1%)
	T3	N = 1 (2.4%)
	T2	N = 3 (7.1%)
	T1	N = 1 (2.4%)
Site	Head	N = 36 (86.0%)
	Body	N = 5 (12.0%)
	Tail	N = 1 (2.0%)
PS (WHO)	0	N = 2 (5.0%)
	1	N = 17 (41.0%)
	2	N = 22 (52.0%)
	3	N = 1 (2.0%)
Previous treatment	None	N = 8 (19.0%)
	Chemotherapy only	N = 23 (54.8%)
	Chemoradiotherapy	N = 4 (9.5%)
	Palliative surgery (gastric/biliary bypass) + chemotherapy	N = 2 (4.8%)
	Curative surgery (Whipples/Resection) + chemotherapy	N = 1 (2.4%)
	Curative surgery (Whipples/Resection) + chemoradiotherapy	N = 4 (9.5%)
SABR purpose	PRIMARY (no other/prior treatment)	N = 8 (19.0%)
	ADJUVANT (within 6 months other primary treatment)	N = 14 (33.3%)
	CONSOLIDATION (> 6 months post-primary treatment to residual disease)	N = 11 (26.2%)
	RETREAT (> 6 months post-primary treatment to disease relapse)	N = 9 (21.4%)

### SABR planning and delivery variables (Table 3)

The majority of patients had 3–4 stably implanted fiducials (64%) and received treatment on 3 consecutive days (88%). PTV size ranged from 15.8–193.6 cc, percentage cover ranged from 79.9–99.5%, treatment duration was 3–7 days, treatment delivery time ranged from 36 to 162 mins, BED ranged from 23.4–79.0 Gy and the mean minimum dose to PTV ranged from 11.07–34.45 Gy. Mean and 95% confidence interval (CI) values are given in Table 2. Alternative planning and motion management strategies (ITV and XSight® Spine) were necessary in two palliative patients.

**Table 3:** SABR planning and delivery variables (N = 42 patients)

Number of fiducials	0	N = 2 (5.0%)*
	1	N = 8 (19.0%)
	2	N = 5 (12.0%)
	3	N = 21 (50.0%)
	4	N = 6 (14.0%)
Treatment duration (days)	3	N = 37 (88.1%)
	4	N = 1 (2.4%)
	5	N = 3 (7.1%)
	7	N = 1 (2.4%)
PET scan data used for planning	Yes	N = 11 (26.0%)
	No	N = 31 (74.0%)
PTV size (cc)	Mean (95% CI)	76.25 (63.83–88.67)
Prescription dose (Gy)	Mean (95% CI)	26.77 (19.33–113.39)
Prescription Isodose (%)	Mean (95% CI)	67.3 (65.24–69.35).
Percentage Cover (%)	Mean (95% CI)	96.6 (95.52–97.66)
Min dose to PTV (Gy)	Mean (95% CI)	22.29 (21.0–23.5)
Max dose to PTV (Gy)	Mean (95% CI)	40.2 (38.5–41.9)
Mean dose to PTV (Gy)	Mean (95% CI)	31.5 (30.8–32.3)
Homogeneity Index (HI)	Mean (95% CI)	1.5 (1.45–1.55)
BED (Gy)	Mean (95% CI)	50.3 (47.7–53.0)
Fraction dose (Gy per fraction)	Mean (95% CI)	8.9 (8.6–9.2)
Delivery time (mins)	Mean (95% CI)	71.4 (65.2–77.6)

*alternative planning and delivery strategy used for 2 patients

### Acute and late treatment toxicity incidence (Table 4)

Acute treatment toxicity information was available in 37 patients. Of these, 11 patients (30%) experienced no adverse effects and 23 patients (62%) experienced Grade 1–2 adverse effects, of which fatigue, nausea and pain were the most common. Only three patients (8%) experienced Grade 3 acute toxicities (pain, fatigue and obstructive jaundice, respectively), which resolved quickly with clinical support. Late toxicity information was available for 32 patients, of whom 27 patients (84%) reported no late adverse events. A total of six patients (19%) experienced late adverse effects. One patient (3%) was diagnosed with Grade 2 pancreatic insufficiency and one patient (3%) experienced Grade 2 pain and nausea. Four patients suffered serious duodenal toxicities attributed to treatment: two patients developed Grade 4 duodenal strictures and two patients suffered Grade 4 gastro-intestinal GI haemorrhage. The patient who experienced Grade 2 pain and nausea also developed Grade 4 GI bleeding at 7 months post-treatment: however, the latter incidence was considered unrelated to treatment as it was concomitant with radiologically confirmed local tumour progression invading the duodenum.

**Table 4:** Acute and late toxicity

	Grade 1–2	Grade 3	Grade 4
Acute (≤ 3 months post-treatment) toxicity incidence in N = 37 patients			
NONE	N = 11 (30%)		
Diarrhoea	N = 5 (14%)	0	0
Nausea	N = 8 (22%)	0	0
Vomiting	N = 2 (5%)	0	0
Dyspepsia	N = 2 (5%)	0	0
Anorexia	N = 2 (5%)	0	0
Pain	N = 7 (19%)	N = 1 (3%)	0
Fatigue	N = 11 (30%)	N = 1 (3%)	0
Jaundice	N = 1 (3%)	0	0
Obstructive Jaundice	0	N = 1 (3%)	0
Late (> 3 months post-treatment) toxicity incidence in *N* = 32			
NONE	N = 27 (84%)		
Pain	N = 1 (3%)*	0	0
Nausea	N = 1 (3%)*	0	0
Pancreatic Insufficiency	N = 1 (3%)	0	0
GI Bleeding	0	0	N = 2 (6%)
Duodenal Stricture	0	0	N = 2 (6%)

*one patient experienced Grade 2 pain and Grade 2 nausea

### Association of SABR planning and delivery variables with toxicity incidence (Tables 5 and 6)

Treatment factors were examined in relation to post-treatment toxicity incidence (Table 5).

**Table 5:** Association of treatment factors and treatment toxicity

	Acute toxicity max grade (n = 37)				Late toxicity max grade (n = 32)			
	0	1–2	3	*p*-value	0	2	4	p-value
Number of fiducials†								
0–1	1	6	0	0.0002*	4	1	2	0.01*
2–4	10	17	3		22	1	2	
Treatment days †								
3	11	20	3	0.37	25	2	2	0.012*
4–7	0	3	0		1	0	2	
Previous surgery†								
Yes	1	5	0	0.47	4	0	1	0.68
No	10	18	3		22	2	3	
Previous irradiation†								
Yes	0	7	1	0.11	6	0	0	0.43
No	11	16	2		20	2	4	
Min dose to PTV†								
< 22.35Gy	5	10	2	0.75	9	2	2	0.17
≥ 22.35Gy	6	11	1		17	0	2	
SABR purpose†								
Sole primary	1	6	0	0.064	6	1	0	0.62
Adjuvant	6	4	1		7	0	1	
Consolidation	4	6	1		8	1	1	
Retreat	0	5	2		5	0	2	
Treatment time (mins)	77.5	69.9	68.3	0.570	67.7	59.5	83.0	0.045*
PTV (cc) ‡	93.5	72.1	67.9	0.355	77.2	61.5	79.3	0.875
PTV min dose (cGy) ‡	2342.6	2227.6	2115.8	0.605	2317.7	1901.5	2332.4	0.396
PTV max dose (cGy) ‡	3951.3	3974.7	4149.2	0.8819	4120.6	3920.5	3861.9	0.622
PTV mean dose (cGy) ‡	3151.9	3118.0	3241.9	0.879	3225.4	3085.6	3015.0	0.493
Prescription dose (cGy) ‡	2736.4	2647.8	2700.0	0.735	2740.4	2700.0	2612.5	0.7067
Prescription isodose (%) ‡	69.6	67.5	65.3	0.554	67.0	69.0	68.3	0.882
Percentage cover (%) ‡	96.1	97.0	97.3	0.771	97.1	88.1	91.1	0.002*
BED (Gy10) ‡	52.3	49.2	52.0	0.650	52.1	51.0	49.0	0.811
HI ‡	1.44	1.50	1.54	0.523	1.51	1.46	1.47	0.833

*Statistically significant at 0.05% level † Numbers are frequencies, tested for association using Chi-squared test; ‡ Numbers are means, tested for association using ANOVA

**Table 6:** Duodenal dosimetry and treatment factors for patients with Grade 3+ toxicity

Grade 3+ Toxicity	Treatment Factors				Duodenal Dosimetry					
	Fids	PTV	Prescribed Dose	Dur’n	D0.035 cc	D1.0 cc	D5.0 cc	D10.0 cc	V15 Gy	V20 Gy
ACUTE										
Grade 3 pain	4	39.84 cc	30.0 Gy 3 fractions	3 days	33.9 Gy*	25.4 Gy	15.8 Gy	12.8 Gy*	5.9 cc	2.5 cc
Grade 3 fatigue	3	44.22 cc	30.0 Gy 3 fractions	3 days	34.9 Gy*	27.9 Gy	20.5 Gy*	16.2 Gy*	12.3 cc	5.4 cc
Grade 3 obstructive jaundice	4	119.57 cc	21.0 Gy 3 fractions	3 days	23.8 Gy*	22.6 Gy	21.1 Gy*	19.6 Gy*	32.2 cc	8.6 cc
LATE										
Grade 4 duodenal stricture	1^†^	20.34 cc	28.5 Gy 3 fractions	5 days	11.2 Gy	8.3 Gy	6.8 Gy	6.2 Gy	0.0 cc	0.0 cc
Grade 4 GI bleed	1^†^	83.49 cc	27.0 Gy 3 fractions	5 days	29.3 Gy*	28.3 Gy	26.2 Gy*	23.9 Gy*	39.1 cc	19.4 cc
Grade 4 GI bleed	3	109.31 cc	22.0 Gy 3 fractions	3 days	24.2 Gy*	23.2 Gy	22.5 Gy*	21.8 Gy*	63.3 cc	24.3 cc
Grade 4 duodenal stricture	3	104.25 cc	27.0 Gy 3 fractions	3 days	31.6 Gy*	29.1 Gy	26.0 Gy*	21.8 Gy*	24.2 cc	12.8 cc

*Duodenal planning dosimetry exceeded institution preferred 3 fraction constraints. † single fiducial conferring anticipated increased risk of duodenal complications [47]

The number of fiducials statistically associated with incidence of acute toxicity (*p* = 0.002). A trend for uneven acute toxicity was observed with treatment purpose: relatively few patients who received only SABR experienced acute toxicity but patients who received SABR for palliative re-treatment of relapsed disease showed increased acute toxicity (*p* = 0.064). SABR variables that associated with late toxicity incidence were number of fiducials, treatment duration, treatment delivery time and percentage coverage (all *p* < 0.05). For the two patients that experienced Grade 2 late toxicity, both had outlying delivery times (58 and 61 mins, respectively). Further, one patient had a very low outlying percentage coverage (79%) and was treated with one implanted fiducial for on-treatment tracking.

Seven patients in total experienced Grade 3+ significant toxicities (Table 6). All three patients who experienced Grade 3 acute toxicities breached preferred duodenal constraints. Of the four patients who experienced late adverse treatment effects of Grade 4 GI bleeding or duodenal stricture, it was noted that all four had T4 tumours, none were planned with PET scan information, and all had outlying or sub-optimal treatment variables. Further, preferred duodenal OAR dose constraints were breached in three of the four patients. The first patient with duodenal toxicity was post-resection (distal pancreatectomy and splenectomy was performed the previous year), had received subsequent adjuvant chemotherapy and was re-treated with SABR with palliative intent for recurrent local disease. The surgery had left the patient with distorted anatomy, an unusually small PTV (20.34 cc) and closely associated OAR. Although the duodenum was within the OAR constraint policy, only one implanted fiducial was possible for tumour tracking and the patient was prescribed treatment in three fractions over a longer treatment duration of 5 days because of anticipated toxicity risk. The second patient with duodenal toxicity also received SABR as a re-treatment with palliative intent for locally relapsed disease, also only had one implanted fiducial for tumour tracking, and was treated in 3 fractions over 5 days. Moreover, retrospective examination of the treatment plan showed that the duodenum was the key OAR, encircling and contacting the PTV more than 270°, and duodenal constraints were considerably exceeded. The third patient with duodenal toxicity received SABR as consolidation treatment for residual disease following chemotherapy > 6 months prior. Their PTV was large (109.3 cc) and although both prescription dose (22 Gy) and BED (38 Gy) were lower than average, preferred duodenal constraints were exceeded. The last patient with duodenal toxicity received SABR adjuvant to primary chemotherapy. The patient had a large PTV (104.5 cc) and again duodenal OAR constraints were exceeded.

### Survival outcomes (Fig. 1) and association with planning and delivery variables

**Fig. 1:**
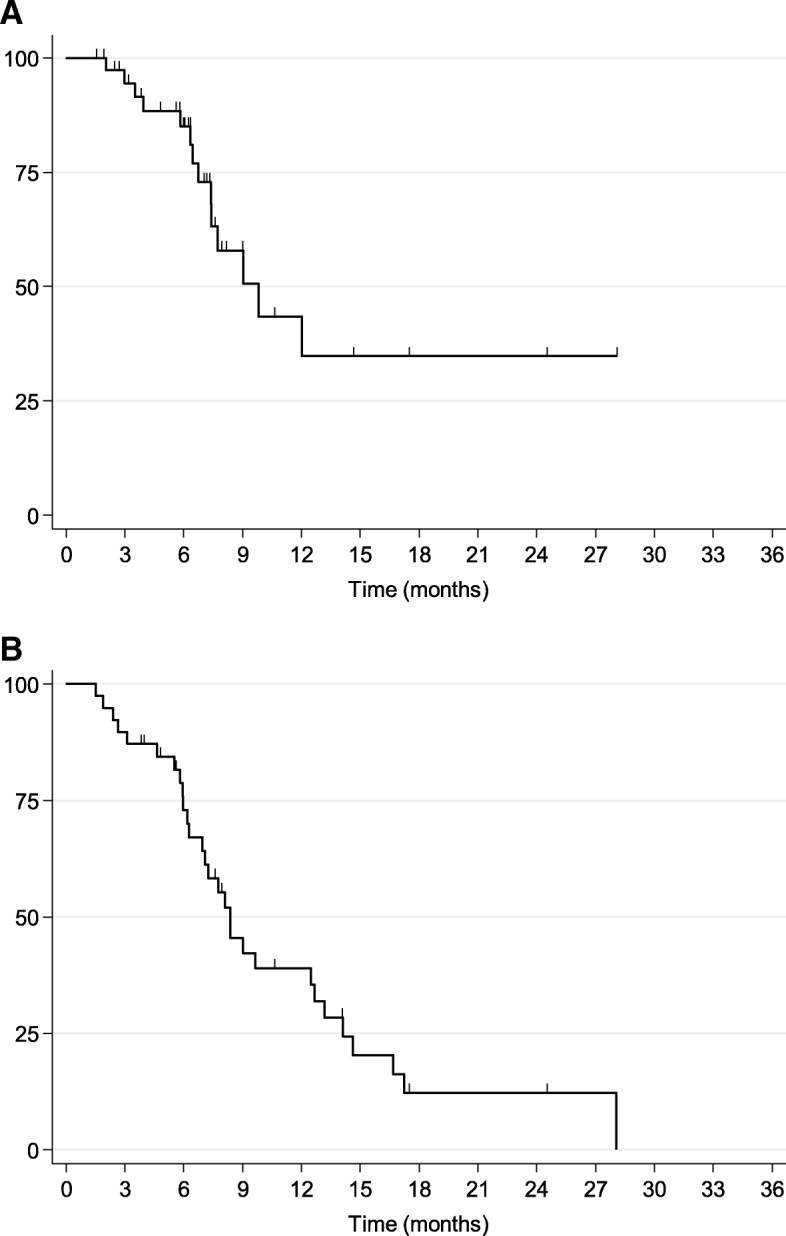
Kaplan-Meier survival plots illustrating **a**) percentage (%) freedom-from-local-progression (FFLP) in months and **b**) percentage (%) overall survival (OS) in months

Post-treatment follow-up information was available for 39/42 (93%) patients. Two patients (5%) converted to resectable status post-treatment: one subsequently underwent complete resection but unfortunately the other was medically unfit for operation. Overall, 15 (36%) patients died with distant progression and no local failure, and two patients (5%) died with local failure and no distant progression. Kaplan-Meier survival analysis showed median FFLP, PFS and OS were 9.8, 5.9 and 8.4 months, respectively. Actuarial 1 year survivor function (95% C.I) for FFLP, PFS and OS were 43.4 (20.8, 64.2)%, 19.8 (7.8, 35.9)%, and 39.0 (22.7, 55.0)%, respectively. Survival plots illustrating FFLP and OS are shown in Fig. 1. Log-rank analysis to test the equality of the Kaplan-Meier survivor function across treatment variables, split by their medians, on FFLP and OS showed no statistical differences. Treatment purpose was also investigated by log-rank analysis, where patients who received SABR > 6 months post-primary therapy as consolidation or re-treat for residual or relapsed disease were compared to patients who received SABR as primary or adjuvant therapy: importantly they showed similar FFLP and OS, indicating SABR may be extending survival outcomes for residual or relapsed disease. When multi-variate Cox proportional hazard regression modelling (adjusted for all treatment variables) was applied to examine the influence of continuous or dichotomous treatment variables on FFLP and OS, both PTV size (cc) and treatment duration (days) significantly associated with OS (*p* < 0.05).

### LQ radiobiological modelling: Comparison of calculated parameters (Table 7)

The BED calculated for the reference conventional conformal radiotherapy schedule (50.4 Gy in 28 fractions delivered in 37 days) was 40.5 Gy_10_. However, as conformal, conventionally fractionated RT is delivered to the minimum 95% isodose within the PTV [45, 46], the minimum dose to the PTV would be 47.88 Gy with the comparator regimen, which equates to a minimum BED of 37.1 Gy_10_. For the normal tissues, the comparator regimen delivers a minimum BED of 75.2 Gy_3._ Using the LQ model, the estimated SABR equivalent to the comparator schedule is 3 × 7.45 Gy, giving a total dose of 22.35 Gy and associated normal tissue BED of 77.85 Gy_3._ For patients in this study who received prescribed dose to optimal individualized isodose (mean = 67.3%), only 21/42 (50%) of patients received a minimum PTV dose of 22.35 Gy.

**Table 7:** Radiobiological (LQ) modelling: calculated comparator regimen and SABR equivalent parameters

	Total Dose (D)	Fraction Dose (d)	Fractions (n)	Duration (days)	BED (Gy_10_)	BED (Gy_3_)
Comparator regimen (100% dose to PTV)	50.4 Gy	1.8 Gy	28	37	40.5 Gy	80.6 Gy
SABR equivalent	23.55 Gy	7.85 Gy	3	3	40.5 Gy	77.0 Gy
	Number of patients treated with total dose ≥ 23.55 Gy = 39/42 (92.9%)					
Comparator regimen (min 95% dose to PTV)	47.88 Gy	1.71 Gy	28	37	37.5 Gy	75.27 Gy
SABR equivalent	22.35 Gy	7.45 Gy	3	3	37.5 Gy	77.85 Gy
	Number of patients treated with minimum PTV dose ≥ 22.35 Gy = 21/42 (50%)					

## Discussion

This study was the first to use LQ modelling to assess pancreatic SABR efficacy and to comprehensively examine the influence of patient and planning factors on toxicity and outcome. The study was conducted in a ‘real-world’ patient series, which is crucial for understanding treatment effectiveness and safety in everyday clinical practice. Our results support SABR as an encouraging modality for locally inoperable pancreatic cancer, with particular benefits as a re-treatment, consolidation or salvage boost, or as an alternative treatment for patients with poor PS or co-morbidity. Although toxicity was low and survival was better than expected — especially considering nearly half of patients were treated more than 6-months after primary treatment — both FFLP survival analysis and LQ modelling showed scope to improve local control through dose escalation. The few serious incidences of late duodenal toxicity indicate that this would need to be approached cautiously with increased stringency on duodenal protection through highly detailed individualised planning.

Low toxicity was obtained that was better than many other SABR studies [20–22, 25, 28, 36], despite larger PTVs and higher prescription doses in this study. This indicates planning methods and on-treatment dynamic targeting with Synchrony® were generally good and consistent with other studies [20, 25, 27–30, 32, 34, 36]. The main limitation when examining patient and treatment factors in relation to toxicity was the low incidence of late and Grade 3+ toxicity events: multiple testing is acknowledged as an additional limitation. The only factors that showed association with acute toxicity were number of fiducials (*p* = 0.0002) and treatment purpose (*p* = 0.064). The former aligns with our previous dose-volume histogram study of duodenal risk in an overlapping cohort of pancreatic SABR patients that showed increased toxicity risk for patients with single or no fiducial [47]. The latter is probably due to the patients who were re-treated who had one or more sub-optimal treatment factors (unusually large or small PTV and/or tightly adjacent OAR and/or only one fiducial and/or breached OAR constraints) and thus a higher risk of treatment toxicity was expected. Number of fiducials also associated with late toxicity [47]. Other variables that associated with late toxicity were increased treatment duration period (> 3 days), treatment delivery time (mins), and coverage: (%): however, we consider these factors to be associative rather than causal for late toxicity for several reasons. First, the main reason for serious duodenal toxicity in three patients was breached duodenal OAR constraints. Second, the uneven distribution noted for treatment delivery time and late treatment toxicities is mainly because two patients who showed Grade 2 late treatment toxicities had lower than average treatment times. Third, the association between treatment duration and percentage coverage likely reflects purposeful selection of these parameters because of recognised increased duodenal toxicity risk due to individual medical, anatomical planning and delivery challenges. For example, SABR is known to be least successful when the treatment volume is large and the ‘dose-cloud’ is not tightly conformal, and to be sub-optimal when fewer fiducials are used compromising the accuracy of dynamic tumour tracking. As such, it is notable that of the four patients that suffered duodenal toxicity, two had large PTV (> 100 cc), and the other two (who were treated palliatively > 6 months after primary therapy for recurrent disease) had only one fiducial implanted for on-treatment tumour tracking and were purposefully prescribed treatment over 5 days because of anticipated toxicity risk. Moreover, three of the patients who suffered duodenal toxicity breached the preferred duodenal OAR dose constraints, with the volume of duodenum receiving > 15 Gy considerably exceeding limits correlated with duodenal toxicity [36]. Increased vigilance and stringency to duodenal dose constraints, particularly for patients with large PTV, distorted anatomy, closely adjacent OAR, or fewer fiducials, is therefore appropriate. Indeed, our recent dose-volume histogram duodenal risk map study of duodenal risk in an overlapping cohort of pancreatic SABR patients showed a 10% risk level for Grade 3–4 duodenal haemorrhage or stricture when D1 cc = D31.4 Gy [47]. Furthermore, we found that the use of multiple fiducials showed ~one-fifth of the risk for Grade 3–4 duodenal complications compared with single fiducial or Xsight® Spine tumour tracking. As such, we advocate implanting at least four fiducials and using three or more stable fiducials for optimal tumour tracking during treatment delivery and, based on estimated 10% duodenal risk levels, now impose the stringent duodenal constraint of D1.0 cc < 31.4 Gy for all pancreatic patients treated with ≤30 Gy in three fractions. Concomitantly, in response to collective experience, we have revised our duodenal D0.035 cc constraint to the higher limit of 24 Gy [47]. It is additionally noted that the incorporation of more extensive measures for duodenal assessment during individualised planning/schedules, such as Lyman modelling for normal tissue complication probability [36], late complications [44] or Red Shell volume calculation [48] may also be helpful in the prevention of duodenal toxicity.

As all patients were inoperable and non-metastatic, they would be expected to have similar prognostic outcomes to intermediate stage patients and may reasonably be compared with similar intermediate stage cohorts in the literature. Overall survival following SABR was good considering that 48% of patients were treated > 6 months after primary therapy, indicating that SABR may be extending survival times for patients with residual or relapsed local disease. The median OS of 8.4 months is comparable to that of first line chemoradiotherapy treatments in randomised LAPC trials [9, 10, 49] and the actuarial 1 year OS of 39% is comparable [29–31, 33] or better than [21, 22, 25, 32] other studies of SABR in LAPC. However, the FFLP of 43% at 1 year is lower in comparison to other SABR studies [20–22, 25–35], where some achieved FFLP rates of 70–94% at 1 year [22, 25, 29, 32]. Although the low FFLP rate is likely to have been negatively influenced by the fact that almost half of patients were treated > 6 months after primary therapy to residual or relapsed disease, it also suggests that higher rates of local control may be achievable with SABR. The lack of any association of treatment factors with FFLP in log-rank or multi-variate analysis indicates that neither current planning or delivery methods are contributing to local failure. Both PTV size and treatment course duration (days) associated with OS. The relationship between size and OS is not clear and may be related to disease advance, and the association with treatment days likely reflects purposeful selection of a longer treatment course for re-treat/palliative/relapse patients with more advanced disease and worse PS.

Although there are limitations to LQ modelling when extrapolating from schedules involving conventional fraction sizes to those involving larger fractions, the results indicated that 50% of the patients treated received a minimum PTV dose that was less potent than a comparator standard conformal radiotherapy regimen. The main reason was because prescribed SABR dose was delivered to a patient individualised isodose line to balance target volume coverage with adjacent OAR risk, whereas conformal RT is delivered to the minimum 95% isodose within the PTV. It is additionally notable that the reference schedule of 50.4 Gy in 28 fractions over 5.5 weeks is associated with a tumor BED of ~ 40.5 Gy. This is considered likely to eradicate only the smallest tumour of moderate radiosensitivity, whereas pancreatic tumours are typically moderately sized and relatively radioresistant. Recent analysis of clinical radiation dose response in pancreatic cancer using LQ modelling has indicated that a BED of ~ 40.5 Gy may achieve > 50% local control [18]. A BED > 70 Gy was calculated to achieve significant tumour response (size reduction consistent with complete or partial response) and thus a schedule of three fractions, each delivering 9.2 Gy, was proposed accordingly for SABR [18]. Recent studies have provided support for the feasibility, similar toxicity and potential increased local control with escalated dose [50]. In our study, the lack of association of toxicity with maximum dose to PTV or prescription dose indicates that a higher overall dose is unlikely to affect toxicity if measures to limit duodenal dose are emphasized. Together these data indicate need and scope to escalate SABR doses to achieve minimum PTV doses > 22.5 Gy *and* optimal BED > 70 Gy for improved efficacy. Our revised duodenal D0.035 dose constraint to the higher value of 24 Gy will allow greater scope for dose escalation. The recent adoption of 5–6 fraction SABR regimens are additionally anticipated to improve toxicity profiles. However, given the ongoing risk for serious duodenal toxicity in a proportion of patients, dose escalation would need to be implemented with great care and awareness of risk for individual patients.

## Conclusions

CyberKnife® treatment was well tolerated and survival in the cohort was encouraging, supporting SABR as a good treatment option in the primary, adjuvant or re-treatment setting. The LQ modelling results and relatively low FFLP indicate scope for dose escalation to improve local control and survival. However, to avoid serious duodenal toxicity a cautious approach needs to be taken, incorporating careful assessment of individual patients for dose escalation suitability and concomitant enhanced stringency on duodenal protection.

## Abbreviations

^18^F-FDG-PET: 18-fluorodeoxyglucose positron emission tomography
BED: Biological effective dose
CI: Confidence interval
CT: Computed tomography
EUS: Endoscopic ultrasound
FFLP: Freedom-from-local-progression
GI: Gastro-intestinal
GTV: Gross target volume
HI: Homogeneity index
ITV: Internal target volume
LAPC: Locally advanced pancreatic cancer
LQ: Linear-quadratic
OAR: Organs-at-risk
OS: Overall survival
PFS: Progression-free-survival
PS: Performance status
PTV: Planning target volume
SABR: Stereotactic ablative radiotherapy
XSS: XSight Spine
